# Fatal infective endocarditis caused by *Aerococcus sanguinicola*: a case report and literature review

**DOI:** 10.1016/j.idcr.2023.e01732

**Published:** 2023-02-28

**Authors:** Raluca Jumatate, Peter Hammarlund, Madlene Holmqvist, Arash Mokhtari, Magnus Rasmussen

**Affiliations:** a Department of Clinical Sciences Lund, Division of Cardiology, Lund University, Lund, Sweden; b Skåne University Hospital, Lund, Sweden; c Department of Cardiology, Helsingborg Hospital, Sweden; d Department of Clinical Sciences Lund, Division of Infection Medicine, Lund University, Lund, Sweden; e Department of Clinical Sciences Lund, Department of Cardiothoracic Surgery, Lund University, Lund, Sweden

**Keywords:** *Aerococcus sanguinicola*, Infective endocarditis, Aortic valve

## Abstract

*Aerococcus sanguinicola* is a bacterium that can cause urinary tract infections and on rare occasions infective endocarditis (IE). The prognosis of IE caused by aerococci is generally favourable despite that the patients are typically old and have multiple comorbidities. Here we report a case of *A. sanguinicola* native valve aortic IE in a 68-year-old man with an underlying urinary tract condition. The infection led to severe aortic valve insufficiency and rapid death before the patient could be subjected to surgery. This demonstrates that IE caused by *A. sanguinicola* can be severe and cause valve destruction. In addition to the case report, we provide a review of the current literature on *A. sanguinicola* IE.

## Introduction

Infective endocarditis (IE) is a potentially fatal condition caused by bacteria that adhere to the heart valves. The bacteria can damage the valves leading to heart failure or form vegetations which can embolize and cause damage to distant organs. *Staphylococcus auereus, viridans streptococci and Enterococcus faecalis* are the most common bacteria that cause IE, but lately aerococci have gained attention as potential IE pathogens [1]. Aerococci have previously often been misidentified, but with the introduction of MALDI-TOF MS for species determination these bacteria have been increasingly identified as causes of both urinary tract infection and bacteremia [2], [3], [4]. Aerococcal invasive infections are almost exclusively found in persons with urinary tract abnormalities and typically occur in older males [3], [5], [6]. A small proportion of patients with aerococcal bacteremia have IE and these patients typically develop subacute endocarditis with a relatively favourable prognosis [1], [7]. The most common aerococcal species to cause infection in humans is *Aerococcus urinae* whereas *Aerococcus sanguinicola* is a less commonly identified pathogen [3], [4], [7], [8], [9], [10]. In fact, only a few cases of severe infections, including IE, have been reported to be caused by *A. sanguinicola* [1], [11], [12]. Here we describe a case of fatal IE caused by *A. sanguinicola* and review the reported cases of *A. sanguinicola* IE.

## Case report

A 68-year-old male presented to the emergency department with a seven-day history of shortness of breath, upper respiratory symptoms and fever. The symptoms exacerbated in two days prior to admission and progressed rapidly. His past medical history included former smoking, hypertension and obesity (BMI 37.6 kg/m^2^). He had also undergone a total penile and scrotum amputation with a perineal urostomy due to penis cancer six years ago. No relapse was observed.

On admission, he was found to be dyspnoeic and drowsy. His body temperature was 37.6° Celsius. His blood pressure was 180/100 mmHg, with a regular heart rate of 114 beats per minute, a respiratory rate of 40 breaths per minute and oxygen saturation of 92% on 10 litres per minute of supplemental oxygen. No heart murmurs were detected. Bilateral wheezing was heard on lung auscultation and bedside lung ultrasound revealed bilateral B-lines. The electrocardiogram showed sinus tachycardia and widespread ST segment depression. Laboratory studies demonstrated leucocytosis of 30 × 10^6^/mL (3.5–8.8 ×10^6^/mL), haemoglobin of 135 g/L (134–170 g/L), and platelet count of 570 × 10^6^/mL (145–348 ×10^6^/mL). Other laboratory abnormalities included serum sodium of 130 mmol/L (137–145 mmol/L), and serum potassium of 4.7 mmol/L (3.5–4.4 mmol/L). Serum creatinine was 81 μmol/L (60–105 μmol/L), lactic acid was 2.9 mmol/L (0.5–2.2 mmol/L), high sensitivity troponin I was 627 ng/L (<54 ng/L) and the C-reactive protein (CRP) was elevated at 205 mg/L (<5 mg/L). PCR tests for SARS-CoV-2 and influenza were negative. Blood cultures were secured. No urine sample could be collected prior to the administration of antibiotics. Initially, the patient could urinate, but three hours after admission he experienced difficulties and received a urinary catheter, which drained clear urine.

**Fig. 1:**
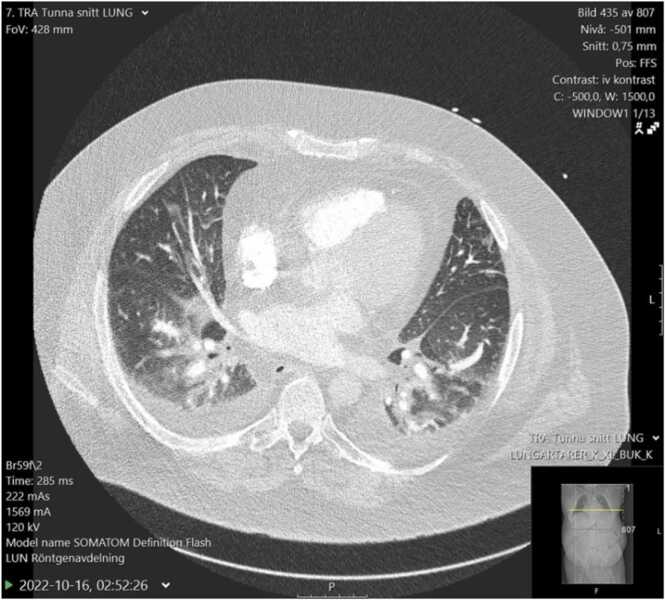
CT scan with signs of pulmonary congestion, bilateral pleural effusion and bilateral peribronchial parenchymal infiltrates.

A transthoracic echocardiogram (TTE) was performed in the emergency department. The quality was not optimal due to poor echo window. The left and right ventricular ejection fractions were assessed as normal. Hypokinesia of the lateral myocardial wall was described. No pericardial effusion was present. Computed tomography (CT) scan revealed no pulmonary embolism. There were signs of pulmonary congestion and bilateral peribronchial parenchymal infiltrates compatible with infection (Fig. 1).

The initial management of the patient was focused on clinical left ventricular failure associated with bilateral pneumonia. The aim was to decrease pulmonary congestion, to reverse the hypoxia and thus avoiding endotracheal intubation. Bilevel positive airway pressure was applied and loop diuretics were given. This led to improvement of the respiratory function. He was transferred to the cardiac intensive care unit and his state improved. Cefotaxime 1 g every 8 h and levofloxacin 500 mg every 12 h were administered intravenously on the suspicion of severe community acquired pneumonia.

**Fig. 2:**
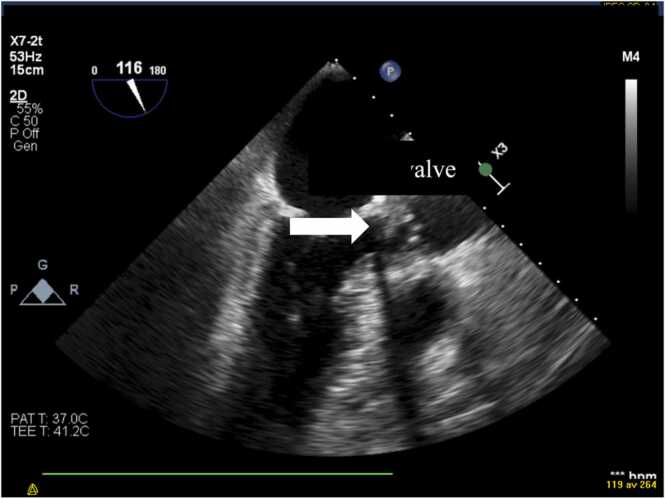
TEE showing a 12 mm mobile mass attached to noncoronary cusp of the aortic valve, protruding into the left ventricular outflow tract.

**Fig. 3:**
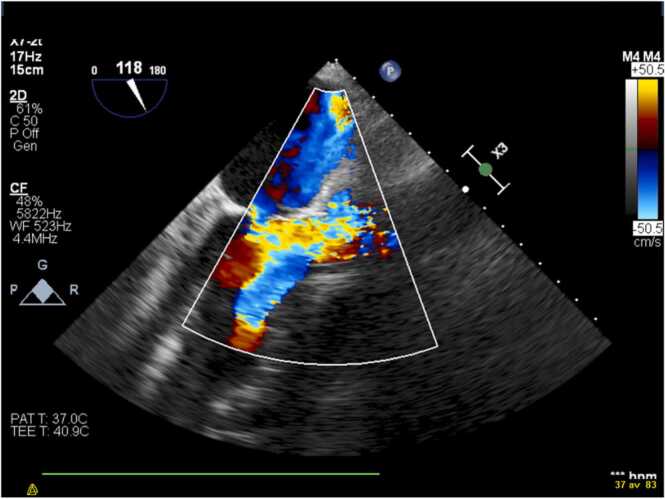
Severe aortic regurgitation due to infective endocarditis of the aortic valve.

**Fig. 4:**
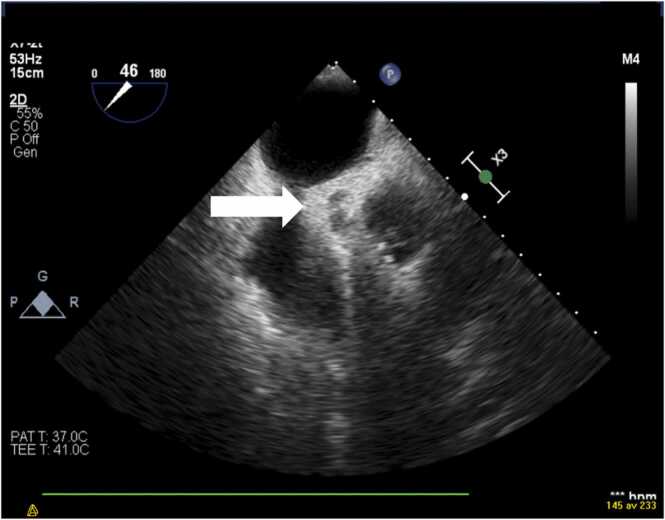
A perivalvular echolucent area compatible with an aortic root abscess.

A comprehensive bedside TTE and a transoesophageal echocardiography (TEE) were performed 12 h after admission. The left ventricle was normal in size with discrete apical and lateral hypokinesia. The left ventricular ejection fraction was normal. The size and the systolic function of the right ventricle were in normal ranges. The aortic valve was tricuspid. There was a 12 mm highly mobile mass on the non-coronary cusp of the aortic valve (Fig. 2) with severe aortic regurgitation (Fig. 3). A perivalvular echolucent area suggesting an aortic root abscess was observed (Fig. 4). No signs of endocarditis on the other valves were detected.

The hemodynamics of the patient remained stable for the next few hours. Peripheral oxygen saturation was 95% with 3 litres per minutes of oxygen without need for non-invasive respiratory support. The heart rate was 100/min. Blood pressure was 130/70 mmHg. The dose of cefotaxime was increased to 3 g every 8 h. As the patient had been temporary stabilized, a multidisciplinary decision was made by cardiologists and a cardiac surgeon to perform coronary angiography prior to an emergency aortic homograft implantation within the next 24 h.

The high sensitivity troponin I continued to rise to a level of 6912 ng/L (<54 ng/L). Since it was deemed preferable to know the coronary anatomy prior to aortic root replacement it was decided to perform a preoperative coronary angiography as soon as possible. Coronary angiography was performed and revealed a significant stenosis in the left anterior descending artery. During the coronary angiography the state of the patient worsened rapidly. The patient was transferred to the cardiac intensive care unit. He developed a fulminant pulmonary oedema and cardiogenic shock with cardiac arrest within a few minutes. Cardiopulmonary resuscitation was attempted but no return of spontaneous circulation occurred. No autopsy was performed.

The next day, blood cultures demonstrated growth of *A. sanguinicola* in all four bottles. Species determination was performed using (MALDI-TOF MS) (Microflex, Bruker Daltonics, Bremen, Germany) with a score value of 2.4. The isolate displayed a minimal inhibitory concentration of 0.125 mg/L for cefotaxime, determined with Etest (bioMérieux). Species specific breakpoints are no available for cefotaxime and *A. sanguinicola*, but according to EUCAST non-species specific breakpoints the isolate was deemed sensitive to cefotaxime.

## Discussion

*A. sanguinicola* is an uncommon cause of bacteremia and IE. Specifically, IE appears to occur in only a small proportion of patients with *A. sanguinicola* bacteremia. In three recent studies of *A. sanguinicola* bacteremia no case of IE was found among 94 patients [3], [7], [10]. The six cases of *A. sanguinicola* IE previously reported are summarized in Table 1.

**Table 1:** Summary of the clinical features of patients with IE caused by *A. sanguinicola*.

Sex	Age	Previous conditions	Location of IE	Antibiotic	Surgery	Outcome	Ref
M^1^	55	Mental retardation	NK	PcG, Ceft+ Gen	NK	Recovered, but died after 3 months	Ibler
F	85	Trisomy 21	NK	Ceft	NK	Recovered	Ibler
M	85	Prostate cancer, dementia	NK	Cefo, Cefo+ Van, PcG	No	Recovered	Senneby
M	65	Trisomy 21	NK	Cefo+Mer, PcG+ Gen	No	Recovered	Senneby
M	54	Diabetes mellitus, skin blisters	AV	Van + Gen	No	Recovered	Sunner-hagen
M	55	Diabetes, recent cystitis	MV	Van+Cefe+ Gen	MVR	Recovered	Abstract Shankar
M	68	Obesity, penile cancer	AV	Cefo+Lev	No	Died	This report

^1^M, male; NK, not known; PcG, penicillin G; Ceft, ceftriaxone; Gen, gentamicin; Cefo, cefotaxime; Van, vancomycin; Cefe, cefepime; Lev, levofloxacin; Mer, meropenem; PAV, prosthetic aortic valve; AV, aortic valve; MV, mitral valve; MVR, mitral valve replacement.

In larger case series describing the prognosis of IE caused by aerococci it appears that the prognosis is relatively favourable despite that patients are typically very old and have multiple comorbidities [1], [3]. In this respect our case is unusual as the patient had an unfavourable outcome despite that he was not very old and did not have multiple known comorbidities. The death was likely caused by the acute IE of the aortic valve and aortic root. The severe acute aortic regurgitation probably led to volume overload of the left ventricle and pulmonary congestion. It also, probably, led to severely decreased stroke volume and coronary perfusion gradients, thus, ending with cardiac arrest due to extensive myocardial ischemia.

The point of entry of the *A. sanguinicola* isolate was not identified in our case but we speculate that the urological problems posed after penectomy was the cause of infection and the point of entry for the bacteria. In patients with underlying urological problems, aerococci are likely among the more common causes of IE. In our case, empirical treatment effective against *A. sanguinicola* was rapidly instituted and therefore the exact aetiology of the IE was not important for the choice of therapy in this specific case.

The indication for acute surgery in this case was clear, based on acute left ventricular volume overload due to acute aortic regurgitation, congestive left heart failure, periannular extension with abscess root formation and a large vegetation. As the state of the patient was temporarily stable and the planned surgical procedure was complex and associated with a high intraoperative risk, the surgical intervention was planned within 24 h, in accordance with current guidelines [13], under careful clinical monitoring.

Current guidelines recommend preoperative coronary angiography in male patients with atherosclerotic risk factors, and in patients presenting with suspected myocardial ischemia, as in our case [14]. Soon after coronary angiography the patient developed refractory cardiogenic shock and cardiac arrest with a fatal outcome.

This case illustrates the well-known high mortality risk associated with acute severe aortic regurgitation due to IE. Moreover, this case demonstrates that IE caused by *A. sanguinicola* may have a severe course.

## Author statement

Raluca Jumatate did the echocardiography and drafted the case report. Peter Hammarlund treated the patient and provided critical comments to the draft. Madlene Holmqvist participated in the care as an ID-consultant, provided the microbiology details and commented on the draft. Arash Mokthari was the cardiac surgeon involved and gave critical comments especially to the parts concerning surgery. Magnus Rasmussen drafted the introduction and discussion and provided expertise on aerococcal endocarditis. All authors agreed to the submission of the manuscript in the present form.

## Competing interests

The authors have no competing interests.
